# Therapeutic Options in Neuro-Oncology

**DOI:** 10.3390/ijms23105351

**Published:** 2022-05-11

**Authors:** Mariana Afonso, Maria Alexandra Brito

**Affiliations:** 1 Faculty of Pharmacy, Universidade de Lisboa, Av. Prof. Gama Pinto, 1649-003 Lisbon, Portugal; marianafonso1911@gmail.com; 2 Research Institute for Medicines (iMed), Faculty of Pharmacy, Universidade de Lisboa, Av. Prof. Gama Pinto, 1649-003 Lisbon, Portugal

**Keywords:** chloroethyl nitrosoureas, immunotherapy, malignant gliomas, signaling pathway inhibitors, temozolomide, tyrosine kinase receptor inhibitors

## Abstract

One of the biggest challenges in neuro-oncology is understanding the complexity of central nervous system tumors, such as gliomas, in order to develop suitable therapeutics. Conventional therapies in malignant gliomas reconcile surgery and radiotherapy with the use of chemotherapeutic options such as temozolomide, chloroethyl nitrosoureas and the combination therapy of procarbazine, lomustine and vincristine. With the unraveling of deregulated cancer cell signaling pathways, targeted therapies have been developed. The most affected signaling pathways in glioma cells involve tyrosine kinase receptors and their downstream pathways, such as the phosphatidylinositol 3-kinases (PI3K/AKT/mTOR) and mitogen-activated protein kinase pathways (MAPK). MAPK pathway inhibitors include farnesyl transferase inhibitors, Ras kinase inhibitors and mitogen-activated protein extracellular regulated kinase (MEK) inhibitors, while PI3K/AKT/mTOR pathway inhibitors are divided into pan-inhibitors, PI3K/mTOR dual inhibitors and AKT inhibitors. The relevance of the immune system in carcinogenesis has led to the development of immunotherapy, through vaccination, blocking of immune checkpoints, oncolytic viruses, and adoptive immunotherapy using chimeric antigen receptor T cells. In this article we provide a comprehensive review of the signaling pathways underlying malignant transformation, the therapies currently used in the treatment of malignant gliomas and further explore therapies under development, including several ongoing clinical trials.

## 1. Introduction

Neuro-oncology is a branch of medicine that focuses on both primary and metastatic brain tumors, spinal cord disorders and complications concerning the peripheral nervous system [1]. This subspecialty is growing and constantly evolving, as new diagnostic, therapeutic and prognostic factors are being discovered [2].

There are over 100 distinct primary brain tumors, which are divided into non-malignant or benign and malignant tumors [3]. Benign tumors are typically not fast-growing or infiltrative lesions. Usually, they are not problematic; however, they can grow and compress structures causing discomfort. Besides, some types can turn into malign tumors, demanding close monitoring [4]. The most prevalent non-malignant brain tumors are meningiomas, followed by pituitary and nerve sheath tumors. Malignant tumors cells grow uncontrollably and invade other tissues. Approximately 80% of malignant brain tumors are gliomas, which constitute a remarkably diverse group of tumors classified according to their microscopic similarities with glial precursor cell lineages, such as astrocytes, oligodendrocytes, and ependymal cells. Main groups include diffuse gliomas, characterized by extensive infiltrative growth, and non-diffuse (circumscribed) tumors [5,6]. Diffuse gliomas are further subdivided into diffuse astrocytomas, oligodendrogliomas and oligoastrocytomas. Glioblastoma (GBM) is the most frequent and most malignant representative of astrocytomas, leading generally to the death of patients within 14 months post-diagnosis [7]. Within these subgroups, a grade is assigned: grade II (low grade) shows nuclear atypia; grade III (anaplastic) displays increased mitotic activity, and grade IV shows additional microvascular proliferation, necrosis or other molecular findings such as the presence of a *CDKN2A/B* homozygous deletion that results in a worse prognosis [8,9,10]. Moreover, a group of non-diffuse tumors, ependymomas, when in the presence of *RELA* gene fusion, also confers a poor prognosis [8].

During the last decade, the knowledge of molecular alterations in tumors of the central nervous system (CNS) has massively expanded, increasing the number of pathological entities [6,11]. In fact, the 2016 World Health Organization (WHO) classification has brought a new paradigm and a new method, combining histological and molecular features into a diagnosis called ‘integrated diagnosis’ [12,13]. This diagnosis adds a new level of objectivity that has been lacking in the past, improving accuracy in classifying CNS tumors [13,14]. Between the diffuse gliomas and their molecular alterations, more than 90% of oligodendrogliomas show 1p19q codeletion and an *isocitrate dehydrogenase* (*IDH*) mutation, which has become a genetic signature of oligodendroglioma. *Tumor protein p53* (*TP53*) and *alpha thalassemia/mental retardation syndrome X linked* (*ATRX*) are intact in the presence of 1p19q codeletion. Thus, when loss-of-function mutations in *TP53* and *ATRX* occur, the diagnose of oligodendroglioma can be excluded. The majority of astrocytomas include the *IDH*-mutant, have intact 1p/19q (no codeletion) and mutations in *TP53* and *ATRX* [9,13]. More recently, in June 2021, the fifth edition of the WHO classification was published. Major modifications are present in the classification of GBM, that now encompass IDH wildtype diffuse astrocytic tumors in adults. In the absence of the histologic features of GBM, tumors can also be classified as GBM if one or more of three genetic parameters, namely *telomerase reverse transcriptase* (*TERT*) promoter mutation, *epidermal growth factor receptor* (*EGFR*) gene amplification, or combined gain of entire chromosome 7 and loss of entire chromosome 10 [+7/−10]) are present [10].

In this article we review the mechanisms underlying carcinogenesis and provide a comprehensive overview of the therapeutic options in neuro-oncology, ranging from general chemotherapeutic options to promising emerging strategies.

## 2. Carcinogenesis

**Figure 1:**
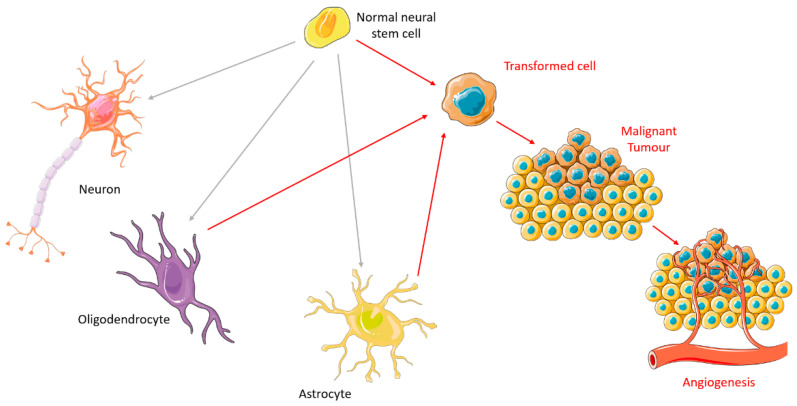
Carcinogenesis in brain cells. Figure drawn with smart.servier.com.

Studies over the past century have helped clarify the basis of tumorigenesis [14]. There are two principal phases: the preliminary phase, characterized by latent intracellular changes, and the phase of clinical symptoms of tumor growth [15]. There are some characteristics of the cancer cell phenotype that result in its progression, such as self-sufficiency in growth signals, insensitivity to antigrowth signals, evading apoptosis, limitless replicative potential, sustained angiogenesis and tissue invasion and metastasis [16]. It is important to recognize that not all cells have the same proliferative potential [17,18]. In brain tumors, those with the greatest ability to contribute to tumorigenesis have similar properties to normal neural stem cells (NSCs) [17,19]. Due to their durability and specific self-renewing properties, they have a great susceptibility to accumulate carcinogenic mutations, becoming the ideal target of the carcinogenic process [18]. As soon as proliferation and growth happen, the oncogenic pathway is activated and the process of forming a malignant tumor begins. NSCs usually give rise to neurons, oligodendrocytes, and astrocytes; so, the place where the mutations occur will determine the type of tumor it forms. When the tumor grows beyond a critical size (1-2 mm in diameter), it requires new blood vessels to ensure vital levels of oxygen and nutrition [20]. Thus, the tumor finds ways to increase its vascular supply so that it can satisfy the demands of the growing population of tumor cells [21]. This occurs primarily via angiogenesis, the process through which new blood vessels are developed from pre-existing vasculature [20,21], as illustrated in Figure 1.

## 3. Tumor Cell Signaling Pathways

**Figure 2:**
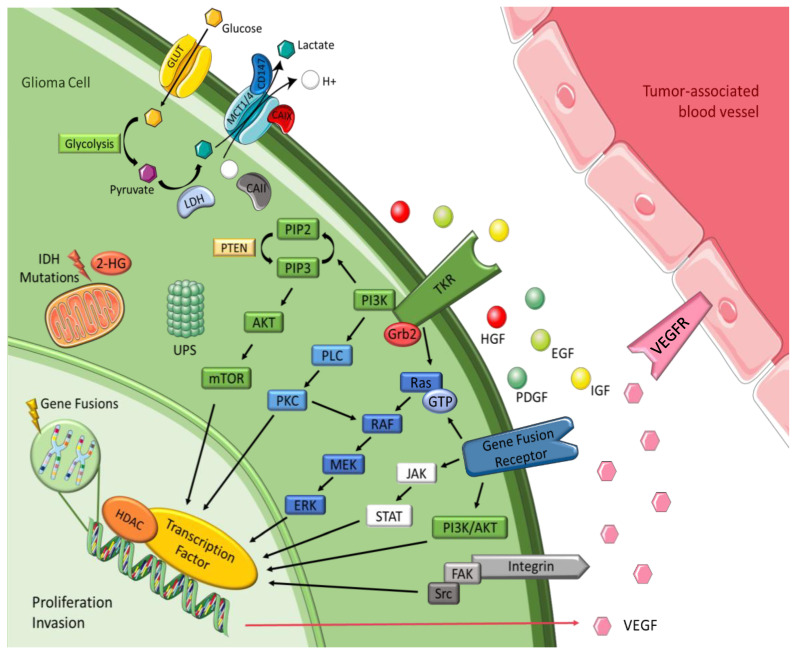
Malignant glioma signaling pathways. Figure drawn with smart.servier.com.

Increasing the understanding of carcinogenesis has allowed the delineation of crucial signaling pathways, which have shown essential roles in the regulation of stem cell functions [2,19]. Accurate and appropriate regulation is doubtless critical for biological activity. However, many of these pathways are dysregulated in cancer, becoming part of oncogenic transformation [19,22]. These transformations involve amplification or overexpression of oncogenes, along with loss or lack of expression of tumor suppressor genes [23]. Therefore, to perceive the logic behind the therapeutic options in gliomas, it is pertinent to understand the different signaling pathways and the transformations in glioma cells outlined in Figure 2 and detailed below.

### 3.1. Tyrosine Kinase Receptor Pathways

Brain tumors cells oversecrete growth factors and overexpress their receptors, creating paracrine and autocrine stimulatory loops, wherein tyrosine kinase is the leading actor [23,24]. These growth factors include epidermal growth factor (EGF), platelet-derived growth factor (PDGF), vascular endothelial growth factor (VEGF), and insulin-like growth factor (IGF). They are correlated with tyrosine kinase and share common mechanisms and intracellular signaling activation [23,25]. *EGFR* gene amplification is the most frequent alteration in GBM, and leads to EGFR protein overexpression, EGFRvIII being the most common mutation [24,25]. This mutation serves as a strong tumor-restricted antigen, as it is expressed in 30-40% of human GBM tumors to enhance glioma cell proliferation, angiogenesis and invasiveness, but is not expressed in healthy tissue [26]. As previously explained, malignant gliomas need extensive vascularity, so they commonly feature high expression of VEGF to allow new blood vessel formation, whereas tumor-associated endothelial cells express the corresponding receptor, vascular endothelial growth factor receptor 2 (VEGFR-2) [25]. Moreover, platelet derived growth factor receptor (PDGFR) overexpression has been observed in gliomas and associated with tumor growth and angiogenesis, leading to malignant progression [24,25]. GBM cell lines exhibit upregulation of insulin-like growth factor-1 receptor (IGF-1R), resulting in proliferative, antiapoptotic and proinvasive effects [27].

### 3.2. Intracellular Downstream Pathways

Most intracellular effectors are serine or threonine kinases, such as rat sarcoma (Ras), phosphoinositide 3-kinase (PI3K) or phospholipase C (PLC), which are recruited to the cell membrane after activation of tyrosine kinase receptor (TKR) and rely on adaptor proteins such as growth factor receptor-bound protein 2 (Grb2) to relay the signals from the cell membrane [25,28]. Gliomas are correlated with either activation of these effector molecules or inactivating mutations of the negative regulators of these kinases, leading in both cases to accentuated effects and dysregulation [25]. Gliomas barely express oncogenic Ras mutations, but they often have increased Ras activity due to a mutation or amplification of upstream positive regulators, such as EGFR and PDGFR [24,25]. The mitogen-activated protein kinase (MAPK) pathway is initiated due to the farnesylation of Ras that is then catalyzed by farnesyltransferase enzyme to activate the rapidly accelerated fibrosarcoma (RAF), mitogen-activated protein extracellular regulated kinase (MEK), extracellular signal-regulated kinases (ERK) to induce the translocation of proteins to the nucleus in order to promote cell cycle progression and anti-apoptosis genes [24,29]. The phosphatidylinositol (3,4,5)-trisphosphate (PIP3)/ protein kinase B (AKT)/ mechanistic target of rapamycin (mTOR) pathway involves TKR and the loss of phosphatase and tensin homolog (PTEN), a negative regulator of these kinases, culminating in deregulation of proliferation, growth, apoptosis, and cytoskeletal rearrangement [24,25]. Protein kinase C (PKC) regulates cell proliferation, invasion and angiogenesis [25]. It is located at the crossroads of multiple signaling pathways and acts like a relay station for signals to the nucleus. PKC is activated by phospholipase-C (PLC), as well as by PI3K, and then transmits signal to the nucleus via the MAPK pathway (mainly) and via the PI3K/AKT pathway [27].

### 3.3. Histones

Histones are basic proteins that order and package DNA into nucleosomes, playing a crucial role in the regulation of gene expression. Histone deacetylases (HDAC) are enzymes responsible for catalyzing the removal of acetyl functional groups from the lysine groups of both histone and nonhistone proteins. Changes in histones affect transcription, repair, and replication, leading to alterations in cell proliferation, survival and differentiation. This is the reason why they have been considered a key target for cancer therapeutics. Some studies have revealed that both genetic and epigenetic mechanisms are significantly deregulated in glioma cells. In particular, modifications in sequence or expression of gene coding for HDACs may contribute to GBM pathogenesis and progression [30,31,32].

### 3.4. Integrins

Integrins represent a family of transmembrane adhesion receptors that integrate signals between cells and the surrounding stroma. They lack intrinsic catalytic activity, implying the presence of external signals for them to be effective. Their effectiveness occurs throughout the activation of integrin-associated proteins and binding of focal adhesions with non-receptor tyrosine kinases, such as focal adhesion kinase (FAK) and Src. Once integrin dimers are formed, downstream signaling pathways are activated, regulating migration, invasion, angiogenesis, and survival. Integrin-encoding genes are rarely mutated in cancers, but deregulation of integrin signaling is quite frequent. Glioma cells invade brain parenchyma preferably along higher rigidity tracts, such as the vascular basement membrane that contains various integrin ligands [33,34].

### 3.5. Ubiquitin-Proteasome System

The ubiquitin-proteasome system (UPS) is a complex and universal protein degradation pathway essential to ensure the balance between cell proliferation and apoptosis [35]. High grade tumors have an inherent potential of escaping cell cycle control mechanisms such as UPS, which results in uncontrolled cell division [27,33]. Hence the importance of these mechanisms being in constant balance and making this a great therapeutic target.

### 3.6. Gene Fusions

Gene fusions are hybrid genes constituted by the combination of the DNA sequences of two genes known to be oncogenic drivers in multiple malignancies. They have the potential to form chimeric proteins with altered functions, and remain an active area of research [36,37]. The Janus kinase (JAK)-STAT pathway initiates transcription of regions of the genome, inducing the expression of anti-apoptotic proteins and other cell cycle regulators, which leads to cellular growth and proliferation [38]. The incidence of gene fusions has been increasingly recognized in GBM, occurring in up to 50% of tumors, with targetable fusions involving a tyrosine kinase domain in approximately 10%. These predominantly include *neurotrophic tyrosine receptor kinase (NTRK), fibroblast growth factor receptor (FGFR)* and *mesenchymal-epithelial transition factor (MET)* fusions [39]. *NTRK* are encoded by three different genes, *NTRK1*, *NTRK2* and *NTRK3*. Genomic rearrangements in *NTRK* genes result in gene fusions and trigger activation of oncogenic TRK signaling [33]. *FGFR* is the most common fusion expressed in GBM, specifically *fibroblast growth factor receptor 3–transforming acidic coiled coil-containing protein 3* (*FGFR3:TACC3*), which is the fusion with relevance as a potential target in multiple cancers, including GBM [33]. *MET* encodes the receptor for hepatocyte growth factor (HGF) and has great importance in the migration and invasiveness of glioma cells, such as in response to irradiation, inhibition of angiogenesis and hypoxia, and has a critical role in therapeutic resistance and recurrence of GBM [28,33,40].

### 3.7. Isocitrate Dehydrogenase

IDH enzymes play essential roles in the Krebs cycle and cellular homeostasis by catalyzing the oxidative decarboxylation of isocitrate. There are three isoforms, with IDH1 in the cytoplasm and peroxisomes and IDH2 and IDH3 in the mitochondrial matrix. Advances in cancer genetics revealed that the genes encoding IDHs are prevalent in human malignancies, including gliomas. One of the consequences of *IDH* mutation is the alteration of enzymatic activity, which leads to the synthesis of 2-hydroxyglutarate (2-HG) and has been implicated in epigenetic mechanisms of oncogenesis. 2-HG elevation suppresses the electron transport chain, interfering with cellular metabolism and epigenetic regulation. However, the tumor-initiating and progressing capacity of 2-HG had not been fully demonstrated [41,42,43,44].

### 3.8. Transmembrane Monocarboxylate Transporters

A common phenomenon that characterizes the adaptation of tumor cells is the shift from oxidative phosphorylation to aerobic glycolysis as a main source of ATP. A high rate of glycolysis leads to the overproduction of lactic acid, which is associated with poor prognosis. Glucose enters the cell through the glucose transporter and is converted into pyruvate, which turns into lactate due to lactate dehydrogenase activity [45]. Monocarboxylate metabolites such as l-lactate are transported by the monocarboxylate transporters (MCTs) family members MCT1, MCT2, MCT3 and MCT4 [46]. Therefore, these transporters allow glycolysis to operate at a high speed by mediating the efflux of lactic acid into the extracellular environment, which also helps preventing acidosis, thus playing an important role in pH regulation [46]. Among these MCTs, MCT1 and MCT4 are present in astrocytes and stand out in glioma cells, playing a pivotal role in tumor cell survival by promoting lactate efflux [45,47]. MCT2 is the primary isoform expressed in human GBM and glioma-derived cell lines and is expressed in the neurons [48], which use the lactate produced by astrocytes. In the brain, glycolytic oligodendrocytes and astrocytes export lactate through MCT1 and MCT4 to fuel oxidative neurons expressing MCT2 [46]. It was hypothesized that carbonic anhydrases (CAII/CAIX) functions as a proton-collecting antenna, thereby enhancing the activity of proton-coupled MCTs, while CD147 acts as a chaperone facilitating membrane trafficking of MCT1/4 [49,50]. Accordingly, the plasma membrane localization of MCT1 and MCT4 was shown to be regulated by coexpression with the chaperone protein CD147, which was also upregulated in GBM compared to diffuse astrocytomas and nonneoplastic brain [45].

## 4. Therapeutic Options

Surgery, radiotherapy (RT), and chemotherapy have been longstanding concomitant treatment modalities in the management of brain tumors [11,26]. Although advances in diagnosis and treatment regimens have improved the outcome of patients with brain tumors [11,26], high-grade gliomas remain uncurable [43]. In this section we summarize pharmacologic therapies, addressing classic options and pinpointing new approaches.

For therapy to be effective, it is necessary to overcome several obstacles, including drug resistance mechanisms. Indeed, drug resistance is one of the biggest challenges in cancer therapy, existing across all modes of therapy, including chemotherapy, molecularly targeted therapy and immunotherapy [51]. Drug resistance may be due to the tumor’s high propensity toward immunologic escape [52,53]. Based on the factors involved, resistance can be divided into intrinsic or extrinsic resistance. Intrinsic resistance implies that even before therapy, there are factors present in cancer cells that contribute to the reduction of the effectiveness of cancer therapies [54,55]. These factors may differ from patient to patient and even among tumors in a single patient [51]. Extrinsic or acquired resistance can be developed during the treatment of tumors that were once sensitive to cytotoxic drugs that stop being effective due to diverse adaptive responses such as activation of alternative compensatory signaling pathways [54,55]. Moreover, resistance may depend on genetic instability, mutation evolution and intra-neoplastic diversity [54]. Combining treatments with different mechanisms of action can kill more cancer cells and reduce the opportunity of drug resistance development [51].

In therapies targeting the brain, another difficulty arises due to the existence of the blood-brain barrier (BBB) [39], an interface between the blood circulation and the brain that protects against injury, pathogens and toxins. The BBB is formed by microvascular endothelial cells that differ from those of other tissues due to elaborate junctional complexes, lack of fenestrations and a high expression of efflux transporters, which account for restricted permeability. Moreover, endothelial cells are surrounded by a thick basement membrane, pericytes, and astrocytes end feet, which further contribute to the barrier properties [56,57]. For chemicals to penetrate the BBB they must be small (<400 Da) and lipophilic, otherwise they require active transport mechanisms [58]. Therefore, 98% drugs do not get into the brain, which represents a huge obstacle in the delivery of potential therapeutics to the brain and is a major concern in the management of brain tumors [57,59]. Furthermore, the presence of efflux transporter proteins creates another big hurdle, since even when a drug is able to cross the barrier, it may be pumped out [60].

### 4.1. General Chemotherapeutic Options

This section highlights chemotherapy with alkylating agents since they are the most used drugs in the treatment of neoplastic diseases, and most patients with gliomas receive this therapy at some point in their disease course [61]. The mechanism of action of alkylating agents is based on the induction of DNA alterations through the addition of an alkyl group to the guanine base of the DNA molecule, which causes DNA strand breakage and leads to apoptosis. Even though these drugs have been well known for a long time, the quality of data for individual chemotherapy agents or regimens is poor, and comparison across studies is difficult [39]. It is important to highlight that when it comes to chemotherapy, it is necessary to evaluate the benefit/risk and if the treatment is worth it or not due to potential long-term toxicity [61,62]. This type of analysis differs from patient to patient and within the same patient, depending on their condition at the time of the therapy. The most important classic therapies regarding malignant brain tumors include temozolomide (TMZ), chloroethyl nitrosoureas (CNUs) and the combined therapy procarbazine, lomustine and vincristine (PCV).

#### 4.1.1. Temozolomide

**Figure 3:**
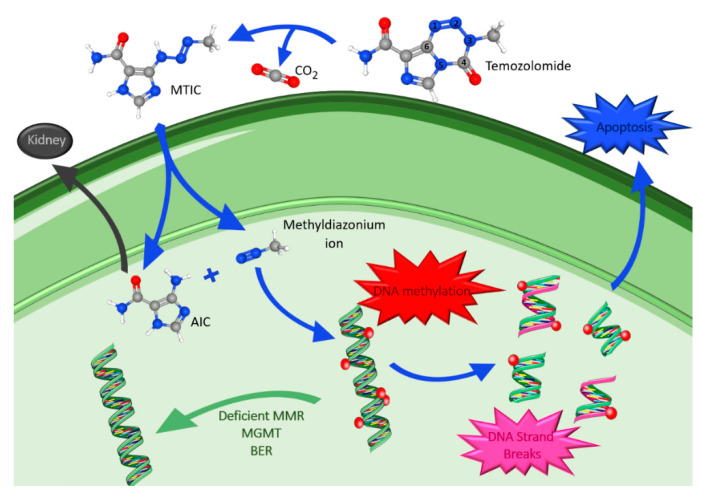
Temozolomide metabolism, mechanism of action and drug resistance. Atom color code: blue, nitrogen; grey, carbon; red, oxygen; white, hydrogen. Figure drawn with smart.servier.com and using molecules from PubChem [68,69,70,71,72].

TMZ is an oral DNA alkylating agent that causes defective DNA repair and cell death, and whose principal mechanism of action is DNA methylation [26,63,64]. It was first synthesized in the early 1980s and approved for medical use in malign gliomas in 1999. Some of its remarkable features are lipophilia and small size (194 Da), which allows passage through the BBB. It is the most commonly used drug in glioma treatment and has been the top choice for many years [62,65]. This drug has a favorable safety profile, with myelosuppression as its dose-limiting toxicity [61]. TMZ spontaneously converts into the reactive methylating agent 5-(3-methyl-1-triazeno)imidazole-4-carboxamide (MTIC) through the effect of water at the highly electropositive C4 position of TMZ at physiological pH, and with release of CO_2_ [63]. In the nucleus of the cancer cell, MTIC is rapidly converted to 5-aminoimidazole-4-carboxamide (AIC) and methyldiazonium ion (Figure 3), thought to be the active alkylating species [66,67].

The methyldiazonium ion transfers a methyl group to DNA producing lesions, mainly at the positions N_7_ and O_6_ of guanine and at the N_3_ of adenine. This causes incorporation of thymine instead of cytosine during DNA replication, creating a mismatched base pair [63,66]. Furthermore, the DNA mismatch repair (MMR) pathway, an enzyme system responsible for the surveillance and correction of errors during DNA replication, repair, and recombination, is activated and tries to repair the damage. However, instead of correcting it, it leads to incorrect DNA crosslinks, DNA breaks and finally to apoptosis [62]. Moreover, the final degradation product, AIC, is excreted via the kidneys [63]. Methyldiazonium ion actions are antagonized by intranuclear DNA repair mechanisms including: O_6_-methylguanine-DNA methyltransferase (MGMT), deficient MMR system and base excision repair (BER). MGMT rapidly reverses modification at the O_6_ position of guanosine, removing the methyl group added by TMZ, and reduces the cytotoxic effects of its action [39]. A deficiency in the MMR pathway allows cell tolerance to methylation and to the cytotoxic effects of TMZ, leading to the continuation past the adducts without cell cycle arrest or apoptosis. Therefore, a good response to TMZ requires functional MMR and low levels of MGMT. Lastly, BER is the major mechanism that repairs the modified DNA components, repairing N_7_- and N_3_-purines, methylated by TMZ [62,65]. The efficient repair minimizes the effect of these lesions and counteracts the action of TMZ. However, if BER is disrupted, the adducts formed become highly cytotoxic, being able to bypass other TMZ-resistance factors [65].

Many clinical trials in gliomas have proved that epigenetic silencing of MGMT, through promoter methylation, leads to increased apoptosis and sensitivity to TMZ [39,62,73]. Furthermore, it is known that TMZ increases poly (ADP-ribose) polymerase (PARP) activity, which is related to BER resistance. Thus, the inhibition of PARP increases the cytotoxicity of methylating agents [63].

TMZ is the standard treatment when there is a poor prognosis, as in astrocytomas WHO grades III and in GBM. TMZ is also a standard treatment at progression after surgery and RT for most patients with *IDH*-mutant gliomas WHO grade II or III [61]. The effect of TMZ is independent of whether the cell is irradiated or not. However, TMZ synergizes with radiation to increase glioma cell death, which leads to many protocols including chemoradiotherapy [62]. Even though TMZ provides a survival benefit in many patients, its myelosuppression toxicity is dose-limiting and new therapies are being developed [74]. The highest tumor-to-blood concentration ratio achieved is <20%, mainly due to BBB. New delivery techniques and more studies are needed to improve this therapeutic effect [59].

#### 4.1.2. Chloroethyl Nitrosoureas

**Figure 4:**
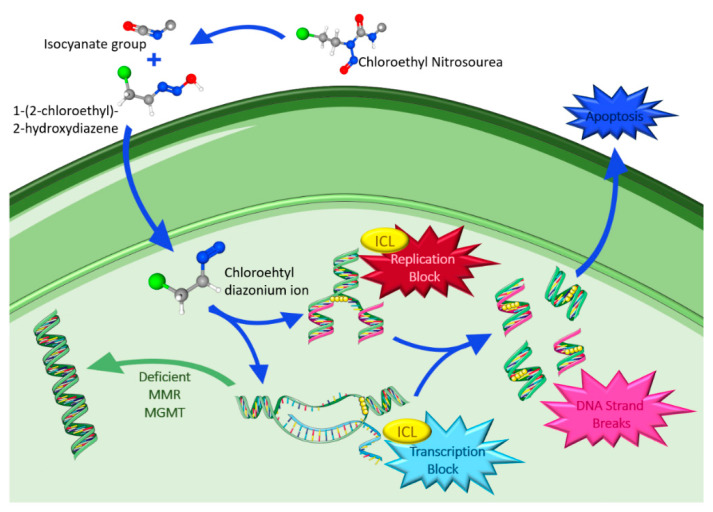
Chloroethyl nitrosoureas metabolism, mechanism of action and drug resistance. Atom color codes: blue, nitrogen; grey, carbon; red, oxygen; white, hydrogen; green, chlorine; grey + white, radical. Figure drawn with smart.servier.com and using molecules from PubChem [78,79,80].

CNUs are DNA alkylating agents that have a long history in the treatment of high grade gliomas and represent one of the most active classes due to their high lipophilicity, which makes it possible for them to cross the BBB. In clinical practice, the most used CNUs are lomustine, carmustine and fotemustine. Their mechanism of action (Figure 4) is characterized by chemical decomposition in aqueous solutions leading to the formation of two reactive intermediates: an isocyanate group and 1-(2-chloroethyl)-2-hydroxydiazene. The latter decomposes into a chloroethyl diazonium ion, which inside the nucleus forms adducts that promote the formation of interstrand crosslinks (ICL), in which the O_6_-chloroethylguanine adduct has been suggested as the major killing lesion [75,76,77].

ICLs are cytotoxic DNA lesions that link the DNA strands covalently and block DNA replication and transcription, resulting in cellular damage and apoptosis. On the other hand, isocyanate does not appear to be directly involved in the antitumor effects of CNU; instead, it inhibits DNA polymerase and other enzymes involved in the repair of DNA lesions and inhibits RNA synthesis and processing, playing a role in the toxicity of the nitrosoureas [81,82]. However, tumor cells may have mechanisms to repair the damage from alkylating agents, such as suicide enzyme MGMT that removes the precursor DNA lesion O_6_-chloroethylguanine prior to its conversion into the ICL. Moreover, in cells lacking MGMT, the crosslink formed stimulates a complex enzymatic network to achieve the removal [82].

Carmustine (BCNU) has important historical relevance, being one of the standard treatments in gliomas in the 1970s and 1980s. However, its use in clinical practice has progressively decreased, and has almost disappeared, due to its particular prolonged bone marrow suppression and persistent lung toxicity [81]. More recently, a biodegradable carmustine wafer has been developed to allow the delivery of high doses of chemotherapy while avoiding systemic toxicity. These wafers contain 3.85% carmustine and up to eight wafers can be implanted into the tumor bed lining at the time of open resection. Clinical trials have shown an overall survival (OS) advantage and defined 65 years old as the patients’ upper age limit [83]. The treatment was approved by the FDA in 1997 for use as an adjunct to surgery to prolong survival in patients with recurrent GBM for whom surgical resection is indicated.

The use of lomustine (CCNU) can be traced back to the 1970s, when the first studies were published, and even though lomustine was more often used, through the years CCNU gradually took place in the treatment of gliomas. Lomustine has the advantage of being an orally administered agent, but it is also characterized by frequent toxicity, such as thrombocytopenia and present dose-limiting pulmonary toxicity [81].

Lastly, fotemustine gained importance later than the previous CNU, especially in Europe, where it is widely used in clinical practice. This is due to its low toxicity in the lungs, which allows a higher number of administrations before reaching its limit toxicity. Its most representative side effect is long lasting thrombocytopenia [81].

In contrast to TMZ, CNU causes delayed and more cumulative leukopenia and thrombocytopenia. Therefore, a closer monitorization is needed, and sometimes treatment interruptions, dose reductions or even discontinuation are required [61]. This, along with the consolidated role of TMZ, led to CNU being mostly used as the control arm in recurrent setting [81].

#### 4.1.3. Polychemotherapy

The combined administration of cytotoxic agents has emerged from the need to overcome the problem of resistance to single-agent chemotherapy [84]. This multidrug regimen kills the cancer cells in more than one way, avoiding cell resistance and being more effective than single agents, becoming the new paradigm for cancer therapy [85]. However, polychemotherapy is also associated with increased toxicity, and close monitoring is necessary [39,86].

Polychemotherapy worked really well in some forms of cancer, and researchers have studied the possibility of a combined regime for glioma tumors. A combination therapy, composed of two oral alkylating agents (procarbazine and lomustine) that induce DNA alterations, and one IV vinca alkaloid (vincristine) that disrupts microtubule formation and mitosis, known as PCV, has been used [26,85].

Procarbazine was discovered in the late 1950s while searching for monoamine oxidase (MAO) inhibitors [87]. However, researchers found out that it has alkylating properties through inhibition of DNA, RNA and protein synthesis [87,88]. Moreover, it is thought that procarbazine accumulates in cancerous tissue and promotes peroxide and hydroperoxide radical formation, imitating the effect of ionizing radiation [88].

Vincristine is a complex molecule produced by the leaves of the rosy periwinkle plant Catharanthus roseus (*Vinca rosea*), whose potent cytotoxicity was discovered in 1958 [88,89]. It was introduced in cancer chemotherapy in the late 1960s and remains in widespread clinical uses [89]. Vincristine biological activity is explained by its specific, fast, and reversible binding to the β subunit of tubulin dimers in a region called the Vince domain. This binding leads to a conformational change in tubulin, increasing its affinity for itself and provoking the formation of paracrystalline aggregates [89]. This phenomenon results in microtubule depolymerization and destruction of the mitotic spindles, causing blockage of dividing cells at metaphase with the chromosomes condensed. Moreover, it inhibits assembly of microtubules, as well as synthesis of nucleic acids and proteins [88].

After many studies, it was concluded that patients who derived the most benefit from combined PCV and RT were patients with 1p/19q codeleted tumors and those with *IDH1* mutations, leading to an improvement of OS [89,90]. According to the 2020 European Association of Neuro-Oncology (EANO) guidelines, PCV is the standard of care for *IDH*-mutant and 1p/19q codeleted oligodendroglioma WHO grade II and grade III, and for *IDH*-mutant astrocytomas WHO grade II [61,91]. In recent studies, it was proved that RT + PCV significantly improved progression free survival (PFS) compared to RT + TMZ for *IDH*-mutant, although RT+TMZ was better tolerated [92,93].

Unfortunately, the successes achieved by polychemotherapy has reached a plateau, and PCV is no exception, leading to the relentless search for new innovative therapies [84].

### 4.2. Targeted Therapies

**Figure 5:**
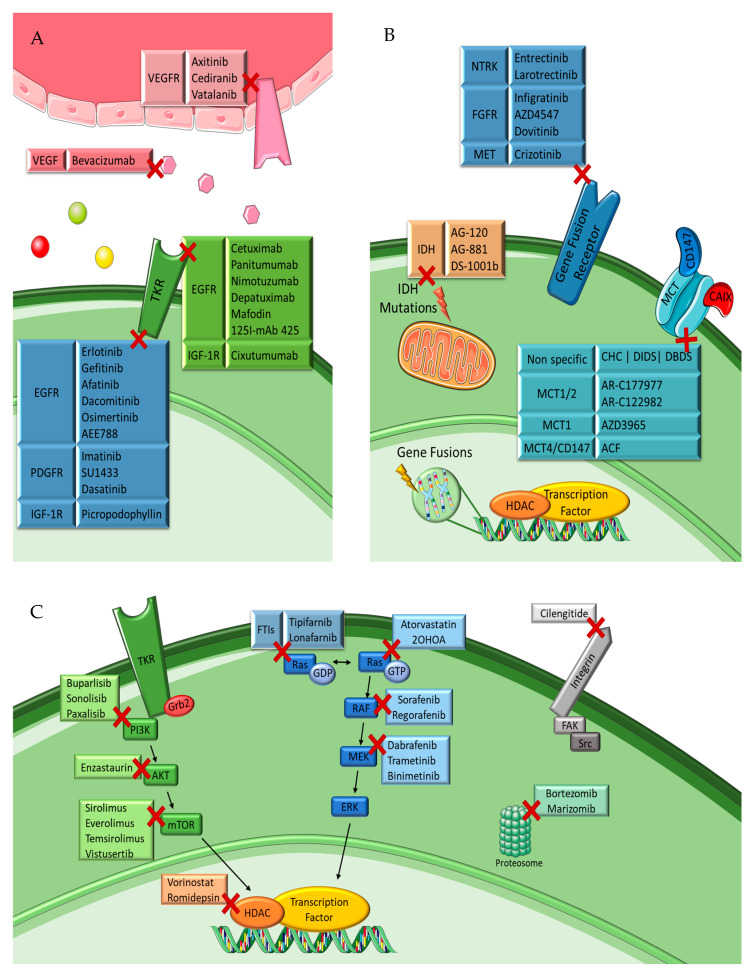
Targeted therapies for malignant glioma. Simplified representation of (**A**) tyrosine kinase receptor inhibitors, (**B**) IDH, gene fusion and MCT inhibitors and (**C**) specific inhibitors of intracellular downstream pathways. Figure drawn with smart.servier.com.

In past years, a lot of progress has been made in the development of targeted therapies, profiting from in-depth knowledge about overexpressed receptors and activated signaling pathways. Clinical success was achieved in several cancers, but there is a constant failure with gliomas [39,84,94,95]. A big hurdle is the BBB, preventing most drugs from reaching their targets in the brain [95]. The preclinical models used to evaluate the efficacy of targeted agents for gliomas cannot effectively address if a targeted therapeutic is truly brain penetrant or not [95]. Targeted therapies have had exponential advances due to the modifications in 2016 WHO classification along with evolution in the understanding of molecular abnormalities of cancers, including brain tumors. These improvements introduced novel insights into cancer biology and created a new world of potential therapies [27,29]. This section focuses on recent advances and on the current clinical trials in targeted therapies, which divide into TKR inhibitors, downstream pathway inhibitors, gene fusions, IDH inhibitors, and MCTs inhibitors. These therapies are schematically represented in Figure 5.

#### 4.2.1. Tyrosine Kinase Receptor Inhibitors

##### EGFR Inhibitors

There are two main classes of EGFR inhibitors: small molecules inhibitors (SMIs) that target the EGFR catalytic domain, and monoclonal antibody-based drugs (mAbs) that target the EGFR extracellular domain [94,96]. The first generation of SMIs includes erlotinib and gefitinib, which presented promising results against GBM cell lines in preclinical studies, but disappointment in following trials both as single agents and in monotherapy [97,98,99,100,101,102]. The second-generation irreversible inhibitors comprise afatinib and dacomitinib. Afatinib showed anti-GBM effects, but only when combined with TMZ in preclinical models, and a pulsatile afatinib administration was demonstrated to be safe and tolerable for patients with brain cancers in a phase 1 clinical trial [103,104]. In more recent studies, dacomitinib showed clinical benefits [105]. The third-generation blockers include AEE788, AZD9291 (osimertinib) and HS-10296 (almonertinib). AEE788 showed inferior brain penetration compared to other EGFR inhibitors such as dacomitinib [106]. Osimertinib demonstrated excellent BBB penetration, with significant exposure in the brain compared to other EGFR-tyrosine kinase inhibitors (TKIs) tested [107]. Moreover, it proved to have a better activity and selectivity for GBM than previous inhibitors [108]. All these features led to promising results in preclinical GBM models, both in EGFRvIII-positive GBM cells with high activity and in EGFR-negative GBM cells, by targeting MAPK-interacting kinases (MNK1/2) [108,109]. Almonertinib is an analog of osimertinib that also showed the capacity to easily penetrate the BBB, which indicates the potential for glioma therapy. Accordingly, the use of almonertinib in non-small cell lung cancer (NSCLC) brain and spinal cord metastases showed promising results in preclinical studies [110,111]. A fourth generation of SMIs (BDTX-1535 and WSD0922) was developed. Patients with glioma or solid tumors (i.e., NSCLC) that metastasize to the brain are presently being recruited to initiate the first clinical trials regarding these two novel molecules (NCT05256290 and NCT04197934). BDTX-1535 is a selective, irreversible inhibitor of allosteric EGFR alterations, which was designed to penetrate the BBB. It has shown activity in preclinical studies against multiple EGFR mutations that occur in GBM [112]. WSD0922-FU is a selective EGFR inhibitor that displays potent activity, excellent CNS penetration, a good safety profile and preclinical anti-tumor efficacy [113]. Although results have not been disclosed yet, these novel inhibitors may constitute promising and cheap options in the treatment of GBM.

Antibodies targeting the L2 domain of EGFR, such as cetuximab, panitumumab and nimotuzumab, were thought to prevent ligand binding and dimerization of this receptor, but both cetuximab and panitumumab had low effectiveness in clinical trials [114,115,116]. In contrast, nimotuzumab showed promising results in preclinical and clinical trials [117,118,119,120]. Antibodies can be conjugated with toxins or radioisotopes to increase their activity [121]. Depatuximab mafodin (ABT-414), an antibody drug conjugate (ADC) composed of the anti-microtubule agent monomethyl auristatin F (MMAF) linked to ABT-806 (a monoclonal antibody that targets EGFRvIII), failed to present survival benefit in newly diagnosed GBM, but when in combination with TMZ in EGFR-recurrent GBM showed a possible efficacy [122]. ^125^I-mAb 425 is an IgG2a isotype developed from mice immunized with A-431 epidermoid carcinoma cells which went through a phase II study that prolonged survival in GBM patients [123]. Finally, AMG595, composed of the maytansinoid DM1 attached to a highly selective anti-EGFRvIII antibody via a non-cleavable linker [122], was developed to treat EGFRvIII-positive GBM patients and showed promising results [124,125].

Despite the failures in the treatment of gliomas with EGFR inhibitors, EGFR remains an attractive molecular target and marker of distinct biologic subtypes. Other approaches, such as combinatorial therapies and personalized therapeutic strategies, are ongoing [126].

##### VEGFR Inhibitors

Malignant gliomas, and specially GBM, are some of the most vascularized tumors, where VEGFR and their ligands play a crucial role as potential therapeutic targets [125]. Therefore, many studies have been conducted to investigate the efficacy of SMIs targeting the VEGFR family. One example is axitinib (AG-013736), a selective inhibitor of VEGFR1-3, PDGFR and c-KIT, which showed good single-agent efficacy with a manageable toxicity profile in patients with recurrent GBM [127,128,129]. However, the association of axitinib with avelumab, a programmed cell death ligand 1 blocking monoclonal antibody, did not reach a synergistic activity against recurrent GBM [130]. Cediranib (AZD2171) is an oral, highly potent VEGFR inhibitor that provided promising results as monotherapy in recurrent GBM [131]. However, it did not show benefit when in combination with lomustine [132]. More recently, it provided benefit survival in combination with gefitinib [133]. There is an ongoing clinical trial to analyze if the addition of cediranib to chemoradiation enhances treatment efficacy (NCT01062425). Vatalanib (PTK787), a VEGFR, PDGFR and c-KIT inhibitor was well tolerated by patients, but did not appear to result in tumor regression [134,135]. There are many other molecules under investigation, but in general there is lack of survival benefit, and more studies are needed.

##### VEGF Trap

VEGF trap are antibodies that prevent the interaction between VEGF and VEGFR, thus avoiding the growth of new blood vessels and inhibiting tumor growth [61,136]. One of the best known is bevacizumab, a mAb recognized by its controversial history. Despite everything pointing to success in early clinical trials, the reality is that no OS benefit has been demonstrated from its use, and increased toxicity occurred, leading to controversy regarding its approval for gliomas [39,61]. Bevacizumab is approved for the use in recurrent GBM in the USA, Canada, and several other countries outside the European Union, but the EMA refuses to extend its indications for treatment of GBM [61,137]. This discrepancy between authorities has led to huge off-label use of bevacizumab in many countries, mostly at recurrence, decided by the Pharmacy and Therapeutics Commission of the hospital, since this antibody is also marketed for the treatment of other cancers [138]. Despite its approval and use off-label, bevacizumab has shown mixed results, whether in monotherapy or in combination [139,140,141,142].

##### PDGFR Inhibitors

PDFGR is overexpressed or amplified in 75% of GBMs and has attracted interest from investigators. Imatinib (Gleevec/ST1571) was the first PDGFR-α, PDGFR-β, BCR-Abl, c-FMS (or colony stimulating factor-1 receptor) and c-KIT tyrosine kinases inhibitor and is responsible for the disruption of the ligand-receptor autocrine loops for PDGFR [96,143]. It showed little beneficial activity for GBM patients in many phase II studies, and in phase III in combination with hydroxyurea [144,145]. Tyrphostin AG1433 (SU1433), a PDGFR-β and VEGFR-2 inhibitor, has shown some promising results, but in further studies its cytotoxic effect was limited. More recently, in combination with RT, it was found that the single treatment was more effective [146,147]. Dasatinib is an inhibitor of PDGFR-β, ephrin type-A receptor 2 (EPHA2), BCR-Abl, c-KIT and Src. Despite upregulation of Src signaling in patients with GBM, dasatinib did not present significantly improved outcomes, either alone or in combination with bevacizumab, or with CCNU in patients with recurrent GBM [148,149].

##### IGF-1R Inhibitors

Picropodophyllin (PPP/AXL1717) interferes with the auto-phosphorylation of IGF-1R. It has shown great results, increasing the radiosensitivity of glioma stem cells (GSCs) and leading to tumor regression in intracranial xenografts, proving its passage through the BBB [150,151]. More recently, a phase I clinical trial demonstrated the capability of AXL1717 to produce prolonged stable disease and survival as a single agent in patients with relapsed malignant astrocytomas. Further investigations will need a new formulation of the drug to better define the optimal dose [152]. Cixutumumab (IMC-A12) was associated with favorable safety and pharmacokinetics profiles [153]. Moreover, its inhibition of GBM growth by different mechanisms was demonstrated, including direct effects on the tumor cells and indirect anti-angiogenic effects [154]. However, a phase I clinical trial combining IMC-A12 with temsirolimus (NCT01182883) in pediatric patients with recurrent or refractory solid tumors, including glioma, was withdrawn.

#### 4.2.2. Intracellular Downstream Pathway Inhibitors

In light of the disappointing activity observed with existing TKR inhibitors, agents designed to interfere with downstream molecules remain attractive [44].

##### Ras/RAF/MEK/ERK Pathway Inhibitors

Rasoveractivation has been highlighted as a target for glioma therapy, but therapies are not able to target Ras directly [29]. Therefore, different classes of Ras antagonists are currently undergoing evaluation, including farnesyltransferase inhibitors (FTIs), Raf kinase inhibitors, and MEK inhibitors [29]. The first therapies targeting Ras were developed against farnesyl transferases once it is known that farnesylation is the rate-limiting step in Ras maturation [27]. FTIs revert cells to a state in which cell-substratum attachment is necessary for viability, leading to apoptosis [143]. Tipifarnib (R115777) can block the prenylation of the farnesyltransferase tail, preventing Ras binding to the membrane and thereby hindering its activation. Tipifarnib has been tested in monotherapy or combined with RT or TMZ or other targeted therapies in many clinical trials (NCT00050986, NCT00058097, NCT00005859 and NCT00335764) [143]. Lonafarnib (SCH66336) has been proven to block cell growth in vitro and in vivo, in combination with chemotherapy and/or RT [155,156]. More recently, lonafarnib was in a phase II clinical trial in combination with TMZ (NCT00038493). Sorafenib is a RAF-1 and p38 inhibitor involved in different pathways, such as Ras-MAPK, VEGFR, c-KIT and PDGFR. Unfortunately, despite promising results in preclinical studies [157], sorafenib did not lead to RT and chemosensitivity in vivo. Several studies led to disappointing results when combining sorafenib with different drugs such as bevacizumab [158], temsirolimus [159,160] or TMZ [161,162,163]. Regorafenib is an orally available multitargeted TKI that emerged from the optimization of the molecular structure of sorafenib, increasing its efficacy [143,164]. It showed an encouraging OS benefit in recurrent GBM in a REGOMA trial (NCT02926222). Some studies and case reports consider regorafenib a new potential treatment for these patients, who are waiting for an adequately powered phase 3 study [165,166]. Moreover, it was realized a real-life study is required to analyse the activity and safety of regorafenib when used as second-line treatment for recurrent GBM patients [167], in line with the results of REGOMA. More recently, a phase 2/3 GBM AGILE study is now recruiting (NCT03970447).

Atorvastatin, a Ras-MAPK and hydroxymethylglutaryl-coenzyme A (HMG-CoA) reductase inhibitor, can increase the effects of TMZ in vivo and in vitro [168]. Moreover, in a phase II clinical trial in newly diagnosed GBM patients, in combination with the Stupp protocol, the standard therapy for GBM consisting of the addition of TMZ to RT, showed promising preliminary results [169]. However, the primary endpoint was not achieved [170]. Nonetheless, this study proved that a high concentration of low-density lipoprotein (LDL) is an important independent predicter of poor cancer-related outcome, and further studies testing statins should aim to enroll patients with slow-growing tumors [170]. Dual inhibition of the MAPK pathway using the BRAF and MEK inhibitors dabrafenib and trametinib, respectively, resulted in durable clinical benefit for patients with *BRAF* V600E mutant low-grade and high-grade glioma according to results from a phase II ROAR study (NCT02034110) virtually presented by Vivek Subbiah, MD, at the AACR Annual Meeting in 2021. In addition, a very recent study included binimetinib (a MEK inhibitor) with encorafenib (a BRAF inhibitor) in adults with recurrent *BRAF* V600-mutated high-grade glioma (NCT03973918).

A lipid proliferation switch led to the development of a new anticancer drug target, the tumor repressor protein sphingomyelin synthase 1 (SGMS1). 2OHOA is a synthetic hydroxylated fatty acid that activates SGMS1, leading to the modulation of the lipid content of cancer cell membranes, regulation of the localization of key signaling proteins, including Ras and PKC at the plasma membrane, and causes the inactivation of Ras/MAPK, PI3K/AKT and PKC/cyclin/cyclin-dependent kinase (CDK) signaling pathways [171]. A clinical trial in Phase I/IIa (NCT01792310) showed its safety and efficacy in humans and, in 2011, the European Commission granted the orphan designation (EU/3/11/ 916) to 2OHOA [172]. Currently, there are two clinical trials ongoing (NCT03867123 and NCT04250922). The latter project, called CLINGLIO, aims at completing a phase IIB clinical trial to prove 2OHOA’s efficacy against GBM. In this context, the EMA wrote a formal report indicating that 2OHOA would obtain Conditional Marketing Authorization if this study further shows statistically significant efficacy [173]. The evolution of RAF/MEK/ERK inhibitors has enhanced targeted cancer therapy and helped in the understanding of molecular mechanisms, which will support the next-generation inhibitors [174].

##### PIP3/AKT/mTOR Pathway Inhibitors

Many PI3K pan-inhibitors have presented promising results, some of which are being tested in clinical trials. Buparlisib (BKM120) inhibits cell invasive capacities in vitro and decreases tumor invasion in vivo [175,176], and is currently being tested in phases I and II clinical trials (NCT01349660 and NCT01339052). In the phase II study, despite good level of brain penetration, there was an incomplete blockade of the pathway leading to bad results [177]. Sonolisib (PX-866) was shown to reduce the invasive and angiogenic properties of GBM cells in vitro and to decrease tumor growth in xenografted mice [178], although phase II studies did not show benefit in recurrent GBM [179]. Paxalisib (GDC-0084) is a potent dual inhibitor of PI3K and mTOR signaling that was tested as a first line therapy for patients with newly diagnosed GBM in a phase 2 study (NCT03522298). According to a press release in December 2021, paxalisib achieved positive results in terms of the drug’s safety and efficacy profile, suggesting a meaningful advantage over TMZ. These data support a GBM AGILE pivotal study that started in January 2021 in which the final clinical data are expected to be published in the first quarter of 2022. Paxilisib was also included in a phase 2/3 GBM AGILE study (NCT03970447), which is now recruiting.

Another target in this pathway is mTOR, wherein the mTOR inhibitors such as sirolimus, and its synthesized analogues everolimus and temsirolimus, have been evaluated in clinical trials of malignant gliomas [27]. Temsirolimus has recently shown the ability to target GSCs, and has become the subject of various studies, in which some did not show clinical benefits [160,180]. More recently, a clinical trial compared the combination of temsirolimus with RT in newly diagnosed patients with the Stupp protocol [181]. It is currently being tested in the N2M2 (NOA-20) clinical trial (NCT03158389). Everolimus presented similar results, wherein a recent study administrating everolimus before the Stupp protocol in newly diagnosed patients did not produce any clinical benefit to the standard protocol [182]. Vistusertib (AZD2014), an inhibitor of both mTORC1 and mTORC2, leads to radio sensitization of GSCs in vitro and in vivo, suggesting its application to GBM therapy. Moreover, it is in an ongoing phase I clinical trial (NCT02619864). Lastly, enzastaurin, an inhibitor of AKT and PKC, has been compared to lomustine in a phase III study. Enzastaurin was well tolerated and had a better hematologic toxicity profile but did not have superior efficacy compared with lomustine in patients with recurrent GBM [183]. In July 2020, enzastaurin was granted Fast Track designation by the FDA for the treatment of newly diagnosed GBM [184]. Moreover, enzastaurin was dosed in a first phase III Study of Newly Diagnosed GBM in combination with TMZ and RT (NCT03776071).

#### 4.2.3. Proteasome Inhibitors

Bortezomib is a first-generation proteasome inhibitor that induces cell-cycle arrest and apoptosis in glioma cell lines [185]. It was found to inhibit glioma growth and to improve TMZ chemotherapy efficacy, probably via down-regulating the FOXM1–Surviving axis, wherein bortezomib might be a promising agent for treating malignant glioma, alone or in combination with TMZ [186]. It was recently demonstrated that bortezomib may serve as a novel therapeutic strategy to enhance the anticancer activity against GBM of an antifungal drug called ciclopirox [187]. Marizomib is a second-generation, irreversible, and brain-penetrant pan-proteasome inhibitor that has been tested in patients with newly diagnosed and recurrent GBM in phase I and phase II clinical trials [188]. Marizomib was administrated in patients with recurrent GBM in a phase I/II clinical trial as a single agent and in combination with bevacizumab (NCT02330562), and in a Phase II study as a single agent and in combination with ABI-009 (NCT03463265). Currently, marizomib is undergoing a phase III clinical trial in newly diagnosed GBM (NCT03345095). Furthermore, it was recently proved to sensitize primary glioma cells to apoptosis induced by a latest-generation TRAIL receptor agonist [189].

#### 4.2.4. Histone Deacetylase Inhibitors

Many studies have tested HDAC inhibitors as monotherapy or in combination in GBM. For example, vorinostat was tested as a monotherapy and in association with bevacizumab and bortezomib, but it did not improve clinical benefit when compared with other therapeutics [190,191]. Romidepsin (FR901228) was also ineffective for patients with recurrent GBM [192].

#### 4.2.5. Integrin Inhibitor

Cilengitide, an intravenous inhibitor of αvβ3 and αvβ5 integrin, demonstrated promising preclinical and phase I efficacy with the Stupp protocol in newly diagnosed patients [27,193,194]. However, in phase II and III clinical trials this combination was not safe nor sufficiently efficient [33,195,196].

#### 4.2.6. Gene Fusions Inhibitors

NTRK inhibitors, such as entrectinib (RXDX-101) and larotrectinib (LOXO-101), received FDA approval for patients with solid tumors harboring *NTRK* fusions, wherein some of those patients had GBM [39]. The prevalence of *NTRK* gene fusions in GBM appears to be low, and more studies are required to conclude if these agents are active in fusion-positive GBM [33,197]. However, the encouraging results obtained support the ongoing development of second-generation *NTRK* inhibitors with lower tendencies to elicit tumor resistance [198,199].

FGFR are amongst the most altered TKRs in GBM, with many of them altered in more than one subtype. Among FGFR1-3 inhibitors, which have been successful in other types of cancer, infigratinib (BGJ398), AZD4547, and dovitinib are examples. AZD4547 is currently undergoing Phase I/II clinical trials (NCT02824133) in GBM patients expressing the *FGFR–TACC* fusion gene. Infigratinib was able to elicit a partial response or stable disease in a third of the cohort, with reversible and manageable adverse effects, in *FGFR*-altered recurrent GBM patients [200].

Emerging lines of evidence have proved that *MET* amplification is involved in crucial parts of glioma cell biology, making it a promising target for therapy [40]. Crizotinib, a c-MET and ALK inhibitor can lead to sensitization of GBM cells to TMZ [201]. It effectively inhibits the proliferation and survival of *MET*-positive GSCs, compared to *MET*-negative GSCs, and apparently prolongs the survival of mice bearing *MET*-positive GSCs [40]. Nevertheless, there have been only few studies to analyze the safety and activity of crizotinib. Crizotinib was evaluated with TMZ and RT for newly diagnosed GBM (NCT02270034), and with dasatinib in pediatric patients with diffuse pontine glioma and high-grade glioma (NCT01644773).

#### 4.2.7. Mutant IDH Enzymes Inhibitors

The potential benefit of IDH inhibitor is controversial, because once epigenetic changes occur in *IDH* mutated tumors, inhibiting mutant *IDH* may be ineffective [91]. Nevertheless, SMIs of mutant IDH enzymes have preliminary reported favorable safety profiles and signs of activity. AG-120 (ivosidenib), an orally bioavailable small-molecule inhibitor, stabilized the growth of lower grade gliomas but did not show significant activity in high grade gliomas [91]. Currently AG-120 is being tested in different studies, including a phase II clinical trial combined with nivolumab in *IDH1* mutant tumors (NCT04056910). Another study evaluated AG120 and AG-881 (vorasidenib), a potent inhibitor of both IDH1 and 2, in a phase I clinical trial, where both drugs demonstrated brain penetrance and a safe and tolerable profile; further results are awaiting (NCT03343197) [202]. AG-881 was chosen as the molecule for a planned phase 3 study in mutant *IDH* low-grade glioma, which is currently ongoing (NCT04164901). DS-1001b was recently evaluated in a phase I clinical trial where the results revealed the efficacy of the BBB-penetrant drug in orthotopic patient-derived xenograft models, providing a preclinical rationale for the clinical testing of DS-1001b in recurrent gliomas [203]. Moreover, in a phase I clinical trial, DS-1001b was well tolerated with favorable brain distribution, and recurrent/progressive *IDH1* mutant glioma patients responded to treatment [203]. Accordingly, further investigation is ongoing to determine the recommended Phase II dose (NCT04458272).

#### 4.2.8. Transmembrane Monocarboxylate Transporters Inhibitors

Since the beginning of studies on MCTs, there have been many compounds reported to be capable of their inhibition. The first inhibitors identified were α-cyano-4-hydroxycinnamate (CHC) and the stilbene disulfonates, such as 4,4′-di-isothiocyanostilbene-2,2′-disulfonate (DIDS) and 4,4′-dibenzamidostilbene-2,2′-disulfonate (DB-DS) [45]. Both were effective at inhibiting lactate transport in vitro, but demonstrated substantial off-target effects, rendering necessary the development of other specific inhibitors. AR-C177977 and AR-C155858 were developed as highly specific MCT1 and MCT2 inhibitors that act by direct binding to transmembrane helices 7-10 intracellularly [47,204]. AR-C177977 was efficacious in GBM cancer stem cells with high MCT1 expression but exhibited low oral bioavailability with a short plasma half-life, limiting its clinical value. AR-C155858 was shown to be effective both in vivo and in vitro, but these effects were reversed upon exogenous or increased endogenous MCT4 expression. AZD3965, an AR-C155858 variant, was developed as a better candidate for clinical use. In addition to having a greater specificity for MCT1 over MCT2, AZD3965 has shown to be effective against different types of cancer [47]. A recently completed phase I clinical trial demonstrated that AZD3965 can be safely administered at 10 mg twice daily in patients with advanced solid tumors (prostate or gastric) or lymphomas (NCT01791595). Acriflavine (ACF), inhibits the function of MCT4 through disruption of the essential MCT4-CD147 interaction. ACF was validated in GBM stem cells, in vitro and in vivo, where it significantly reduced angiogenesis and tumor progression, most effectively under hypoxia [205]. Summarizing, there are a lot of potential in MCT inhibitors; however, there are still no trials for MCTs in gliomas.

### 4.3. Immune Therapies

For many years, the CNS was considered to be isolated immunologically, due to the unique properties of the BBB, the absence of a classic lymphatic drainage system and an apparent immunocompetence of microglia, the resident CNS macrophages [39,74,206]. However, today these ideas have been deconstructed, and it is evident the presence of lymphatic vessels in the CNS, as well as of antigen-presenting cells (APCs) of varied forms such as microglia, macrophages, astrocytes, and dendritic cells (DCs), have roles in immune surveillance [74].

Glioma tissue is formed by cancer cells and infiltrated non-transformed cells, such as microglia and macrophages, which represent 30–50% of the cellular content of these tumors [207]. While macrophages are mostly differentiated from tumor infiltrating monocytes, microglia are derived from erythro-myeloid progenitors, developed in the yolk sac, that migrated into the CNS at the embryonic stage [208]. Glioma associated microglia/macrophages (GAMs) usually have an important influence in the brain, promoting its development and homeostasis. However, it was shown that they can become programmed to produce several factors that cause chronic inflammatory, immunosuppressive, and pro-angiogenic tumoral microenvironment (TME), leading to tumor progression and survival [209,210,211]. Moreover, glioma cells themselves recruit and reprogram GAMs through the secretion of chemokines and other factors, making the communication between tumor and GAMs bidirectional [207,212].

At the moment, several immunotherapies are being developed and tested in patients with gliomas that target both the innate and adaptive immune compartment [213], which is the highlight of this section. These strategies can be divided into vaccine therapies, immune checkpoint blockade, oncolytic viral therapies, and chimeric antigen receptor (CAR) T-cell therapies [39,214].

#### 4.3.1. Vaccine Therapy

**Figure 6:**
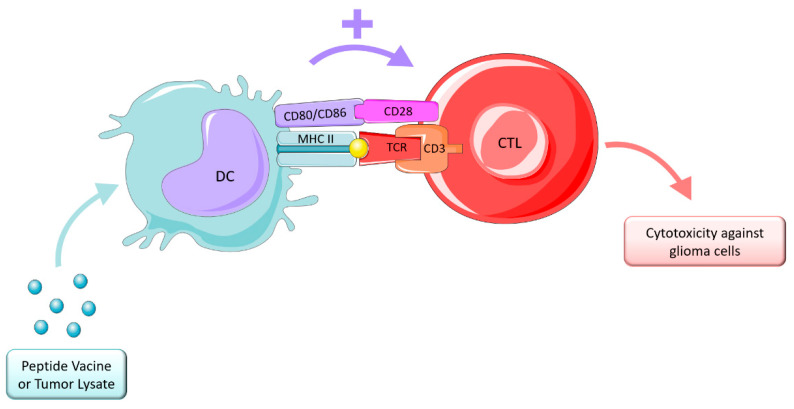
Vaccine therapy. Figure drawn with smart.servier.com.

Cancer vaccine strategies are designed to induce antitumor immune responses which mediate tumor regression by a targeted cytotoxic T-lymphocyte (CTL) effect while avoiding normal tissue [215]. This therapy depends on presentation of peptides, antigens, or epitopes originated from tumor lysates, or autologous or allogenic immune cells being the most common DCs, as depicted in Figure 6. These therapies are divided into two main categories: peptide vaccines and cell vaccines.

Peptide vaccines take advantage of tumor-specific antigens (TSA) to induce an immune response against the tumor cells [215]. The EGFRvIII is the most relevant and uncontroversial TSA for GBM. Peptide vaccines targeting this receptor have been investigated for many years, rindopepimut (CDX-110) being the first vaccine developed against EGFRvIII [206,216]. Early stage trials showed promising results leading to a large randomized, placebo-controlled phase III clinical trial that ended early after showing no significant improvement. However, it was demonstrated that patients developed a decent humoral immune response. Moreover, this study also proved that targeting a single tumor antigen may not be enough to cause favorable responses. IMA950 is a novel vaccine that was designed to target 11 tumor-associated peptides identified on HLA surface receptors in primary human GBM tissue, which can stimulate specific cytotoxic T-cells, leading to destruction of malignant tumor cells [215]. In phase I/II clinical trials, most of the patients with newly diagnosed GBM and other high-grade gliomas responded well. However, as for recurrent GBM, IMA950 showed no benefit in any preclinical trial. IDH1 is highly expressed in low-grade gliomas and in recurrent GBM, where more than 90% of *IDH1* mutations occur at codon 132 containing the R132H mutation. Peptide vaccines covering the *IDH1* mutation might extract IDH1^R132H^-reactive CD4+ and CD8+ responses for antitumor activity. Clinical trials phases I/II are currently on going for IDH1-positive grade II gliomas (NCT02193347) and for high grade gliomas (NCT02454634) and therapeutic results will be soon calculated. Phase III trials are still lacking and are required to support peptide vaccines potential. The single-antigen targeting might not be the strongest option if it leads to antigen escape. Thus, new alternatives are required to target multiple tumor antigens. Heat shock proteins (HSP) vaccines target a mechanism implicated in TSA presentation rather than one antigen, and are a possible solution. HSP-peptide complexes (HSPPCs) mediate endocytosis and stimulate immune responses to tumor-antigenic peptides [215]. HSPPC-96 is a primary resident chaperone of the endoplasmic reticulum that demonstrated promising results in some phase II trials [217,218], but the randomized phase II trial for recurrent GBM failed to pass the interim futility analysis [219,220]. Other clinical trials are currently ongoing in combination with pembrolizumab (NCT03018288).

Cell vaccines involve autologous or allogenic immune cells which cause antitumor immune responses [215]. DCs are the most effective activators of T cell proliferation, so their presence is fundamental for anti-tumor immunity. In order to produce autologous DCs vaccines, first DCs are isolated from the patient, loaded with the tumor antigen, matured via exposure to cytokines and then reinjected into the patient’s body [215,221]. In a phase I trial, Wilms’ tumor 1 (WT1)-pulsed autologous DCs therapy showed safety and immunogenicity in the management of relapsed malignant gliomas [222]. Moreover, cytomegalovirus phosphoprotein 65 RNA (CMV pp65) was also incorporated into DCs vaccines and studied along with TMZ in GBM in a phase I trial, which, despite increased Treg proportions following TMZ, demonstrated long-term PFS and OS [223]. Current ongoing phase II clinical trials await results (NCT02465268). A novel vaccine called DCVax-L was prepared from tumor lysate and has advanced to a phase III trial in which the patients were randomized to TMZ plus DCVax-L or TMZ and placebo after surgery [74]. In this study the secondary endpoint PFS was achieved; however, the primary endpoint was not reported, creating some concerns about this report [215]. Still, the addition of DCVax-L to standard therapy is feasible, safe and might extend survival [224].

#### 4.3.2. Immune Checkpoint Blockade

Nowadays it is well known that there are self-restricting circuits that control activated T cells in order to attenuate the intensity and duration of immune responses, to prevent damage to normal somatic cells [225]. Such circuits include induction of adaptative tolerance in the periphery through the upregulation of ligands of the B7-family, such as CD80, CD86, and programmed death-ligand 1 (PD-L1). They stimulate immune checkpoints, such as activated T cells, to express associated inhibitory cytotoxic T-lymphocyte-associated protein 4 (CTLA-4) and programmed cell death 1 (PD-1) receptors that stop the immune response [226]. However, these mechanisms might be used in favor of cancer cells.

**Figure 7:**
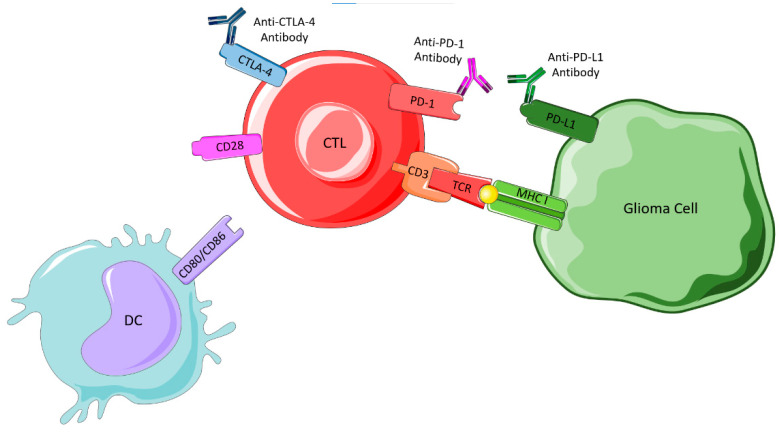
Immune checkpoint blockade. Figure drawn with smart.servier.com.

In glioma cells, the CTLs are activated and destroy tumor cells that present glioma-associated antigens presented on major histocompatibility complex (MHC) class I or II molecules. Tumor cells escape this destruction through the upregulation of the immune checkpoint ligands and presentation of PD-L1 in their membranes, stimulating the immune blockade. CTLA-4 links to CD80 and CD86, preventing their interaction with CD28, a protein required to stimulate T cell activation, and PD-1 binds to PD-L1 to cause suppression of lymphocyte activation. Thus, immune checkpoint inhibitors are required to prevent these interactions, as shown in Figure 7, and allow an immune response.

##### CTLA-4 Inhibitors

Ipilimumab was the first immune checkpoint to be tested and approved in cancer therapy, being also one of the most studied inhibitors [216]. It suppresses T-cell stimulation by competing with CD28 for binding its ligands CD80 and CD86 [206]. In glioma cells, preclinical trials suggest that the blockade could induce tumor regression and promote long-term survival [206]. Some clinical trials are currently ongoing in GBM, testing CTLA-4 inhibitors along with other therapeutics such as VEGF inhibitors (bevacizumab), chemotherapy (TMZ), tumor treating fields and RT [216].

##### PD-1/PD-L1 Interaction Inhibitors

The PD-1/PD-L1 pathway is considered the major negative regulation of CTL in the TME, whose protumor function is inhibiting the secretion of proinflammatory factors and inactivation of T-cell receptor (TCR) signaling [206]. PD-L1 is an immunosuppressive molecule expressed by glioma cells, and evidence correlates the levels of PD-L1 expression with greater invasiveness and aggressiveness, leading to negative outcomes [216,227]. Encouraging preclinical studies triggered the first large phase III clinical trial of PD-1 checkpoint blockade in recurrent GBM by comparing nivolumab monotherapy with standard care using bevacizumab (NCT02017717), but this missed the primary endpoint [206,228]. Phase III studies were conducted, but none of them showed positive results. When nivolumab did not lead to promising results, other therapies emerged, such as pembrolizumab, another anti-PD-1 antibody, which was tested in a phase II trial as neoadjuvant or adjuvant-only therapy with increased OS and PFS. However, when pembrolizumab was compared with concomitant therapy with bevacizumab, the trial did not achieve improved PFS or OS in recurrent GBM [229]. Moreover, durvalumab, a humanized PD-L1 monoclonal antibody was in a phase II clinical trial, combined with bevacizumab and RT (NCT02336165). While the combination with RT showed efficacy among GBM patients with new unmethylated MGMT, the addition of bevacizumab did not improve the outcome of durvalumab alone [230,231]. Currently, atezolizumab is in a phase I/II trial (NCT03174197) in combination with TMZ and RT [215].

In addition to these therapies, other two checkpoint targets have been receiving attention: T-cell immunoglobulin and mucin domain 3 (TIM-3) and indoleamine 2,3-dioxygenase (IDO1). Results from these studies did not meet expectations [206]. Although several checkpoints-related molecules have been discovered, none overcame the influence of PD-1 and CTLA-4, whose efficacy remains to be confirmed [232].

Resistance to immune checkpoint therapy might be related to the lack of penetration of the blocking antibodies, due to their large size, ineffective effector T cell infiltration and/or T cell exhaustion in the TME. Owing to unique resistance mechanisms, monotherapy with immune checkpoints for glioma did not induce antitumor responses. Therefore, a combination of therapies may show a higher efficacy, and chemotherapy, RT, kinase inhibitors and epigenetic drugs may have a synergistic effect on immunotherapy, improving tumor immunogenicity [232,233]. For instance, anti-PD-1 combined with anti-TIM-3 improved survival in preclinical studies [232]. Engineered antibodies with low molecular weight could also be a solution in order to overcome the BBB problem [226].

#### 4.3.3. Oncolytic Viral Therapy

**Figure 8:**
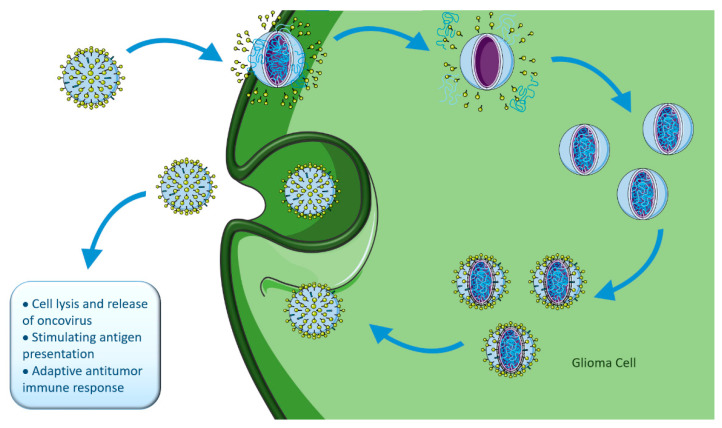
Oncolytic viral therapy. Figure drawn with smart.servier.com.

Tumor regression in patients with concomitant viral infections has been registered [213,234]. This has led to the development of many studies trying to find oncolytic viruses (OVs) that could work as therapeutic strategies for various cancers. OVs selectively infect and replicate in tumor cells, promoting their infection, lysis, and further dissemination of OVs to the neighbor cells. In addition, OVs trigger inflammation and immune responses in uninfected tumor cells, while avoiding normal cells, therefore decreasing collateral toxicity [206,216], as represented in Figure 8.

There are two main types of OVs: viruses non-pathogenic in humans that naturally replicate in cancer cells (e.g., parvoviruses, Newcastle disease virus, reovirus), and viruses genetically manipulated to selectively inhibit their replication in normal cells, but not in cancer cells (e.g., Delta-24-RGD, Toca 511, ONYX-015, PVSRIPO) [216,235]. When the tumor cells die, tumor-associated antigens (TAA) are released into the TME and are recognized by the immune system, stimulating the recruitment of activated immune cells, overcoming the problem of tumor-mediated immunosuppression and activating a systemic response [225]. The interaction between direct tumor lysis and antitumoral immune-response determines OVs effectiveness [236]. There are several early-stage oncolytic viral therapy clinical trials for adult high-grade gliomas with limited, but promising, results [237].

HVS-1-G47-delta is a third generation oncolytic Herpes Simplex Virus type 1, genetically engineered, that was armed with an immunomodulatory cytokine, interleukin (IL)-12, and increased OS in preclinical studies [225,238]. Recent information from a phase II trial where HVS-1-G47-delta was given together with TMZ for recurrent GBM demonstrated a one-year OS higher than the historic control, becoming a preferred treatment that could potentially allow the cure of malignant glioma in the near future [226,239]. Delta-24-RGD (DNX-2401), which is replicating adenoviral vector, showed long term survival in patients with recurrent high-grade gliomas in a clinical trial phase I [240]. This clinical trial also showed that Delta-24-RGD leads to immunogenic tumor cell death and enhancement of T lymphocyte tumor infiltration [216]. In other studies, evidence for elicitation of anti-GBM immune responses (NCT01582516; NCT00805376) was provided [206]. More recently, a phase II clinical trial was completed in which the combination of Delta-24-RGD treatment with pembrolizumab was studied (NCT02798406). It was showed to be well tolerated, with encouraging data emerging for disease control and survival [241]. PVSRIPO (Oncolytic polio: rhinovirus recombinant virus) is a live-attenuated poliovirus type 1 with the internal ribosome entry site replaced with human rhinovirus type 2, allowing the blockage of neuroviolence [242]. PVSRIPO recognizes the poliovirus receptor CD155, present in tumor cells and in major components of the TME, leading to cytotoxicity to tumor cells and activation of an inflammatory response in other tumor cells [216]. Clinical trials phase I (NCT01491893) in recurrent GBM showed promising results and other phase I (NCT01491893) and phase II (NCT02986178) studies are currently ongoing and are necessary to draw conclusive results [39,216]. Lastly, Toca 511 (vocimagene amiretrorepvec) is a non-lytic, retroviral replicating vector that delivers yeast cytosine deaminase, which converts the investigational prodrug Toca FC (5-fluorocytosine) into the antimetabolite 5-fluoracil [216,243]. This conversion induces tumor cell death and depletion of myeloid-derived suppressive cells [244]. A recent phase I clinical trial demonstrated that this treatment led to complete responses and long-term survival. However, a phase III clinical trial for Toca 511 missed the primary endpoint of overall survival compared to standard of care treatment (NCT02414165) [245].

Despite promising results in these therapies, resistance is still a problem and may come from antibodies present in the host’s system that could provoke an immune response against the viral vector, or insufficient diffusion of the viral vectors to the tumor cells. More extensive trials are needed to guarantee the efficacy and safety of these treatments [216].

#### 4.3.4. CAR-T Cell Therapy

**Figure 9:**
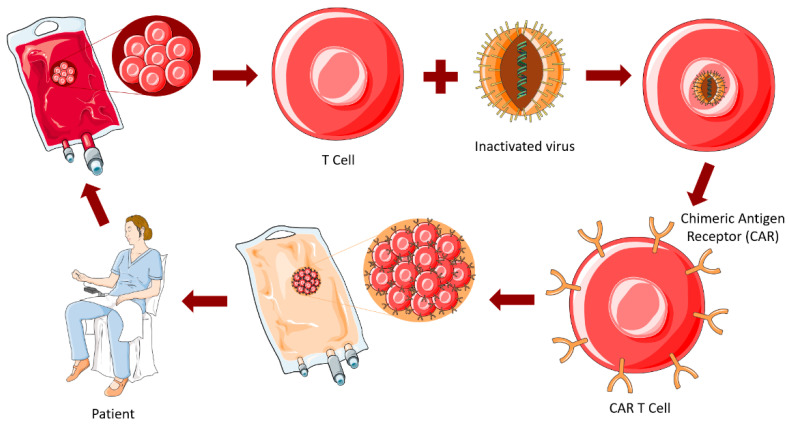
CAR-T cell therapy. Figure drawn with smart.servier.com.

In adoptive T cell therapies, T cells are harvested from patients, and using an inactivated virus new genetic information is introduced into the cell. This way, the T cells are reprogrammed to produce special receptors called CAR, that target specific proteins expressed by the tumor. CARs are artificial fusion proteins that incorporate an intracellular T-cell signaling domain and an extracellular antigen-recognition domain to target specific neoplastic cells. Millions of CAR T (CAR-T) cells are produced in the laboratory and infused back to patients with the goal of destroying tumor cells [246], as represented in Figure 9. This technology transformed immunotherapy and brought in a whole new era. However, difficulties have arisen due to the restrictive properties of the BBB, which limits BBB trafficking of CAR-T cells and their brain access, rendering necessary their administration by invasive methods such as intracavitary or intraventricular infusion [247]. In fact, studies showed that local intracranial and intraventricular delivery of CAR-T cells present superior anti-tumor efficacy as compared to intravenous administration, and trials have focused on local delivery in favor of intravenous delivery [248]. To overcome this limitation, novel therapies are under development, attempting to optimize the delivery, such as BBB disruption through low-intensity pulsed ultrasound, and convection enhanced delivery with hydrogel [249,250].

The CAR-T cells against GBM target EGFRvIII, IL-13Rα2 and human epidermal growth factor receptor 2 (HER2). As mentioned above, EGFRvIII is a receptor that serves as a strong tumor-restricted antigen expressed in 30–40% of human GBM tumors but is not expressed in healthy tissue. IL-13Rα2 is a cancer-germline antigen present in more than 70% of GBM tumors, associated with tumor invasiveness and poor prognosis. HER2 is a receptor usually overexpressed in more than 80% of GBM leading to a bad prognosis [206,246]. Current studies targeting these antigens showed encouraging results regarding safety, feasibility, and penetrance of CAR-T cells into GBM. However, a major difference between CAR-T cells and targeted therapeutic antibody administration is that antibodies undergo clearance by the body, while CAR-T cells can continuously produce effector cytokines and can expand in cell numbers [251]. This might become dangerous, regarding receptors like EGFR and HER2, that are expressed in cardiomyocytes and lung epithelial cells, and are often associated with toxicity in lung and heart. Thus, these therapies require close monitoring in these tissues [252,253,254]. Moreover, findings about the effect on tumor growth and recurrence are less conclusive, and CAR-T cells targeting a single antigen may still lead to antigen escape, so that a lot of effort is being put into next-generation of CAR-T cells, involving multi-antigen targeting, cytokine overexpression and gene editing [216].

Hedge et al. [255] created dual-targeting CAR-T (biCAR-T) cells targeting both HER2 and IL-13Rα2, which could efficiently recognize and kill either HER2 or IL-13Rα2-positive tumor cells, improving the survival while being more sustainable. Later, considering the good results, the same researchers improved the treatment, generating trivalent CAR-T (tri-CAR-T) cells targeting HER2, IL-13Rα2 and ephrin type-A receptor 2 (EPHA2), which overcame the interpatient variability and could capture almost 100% of the tumor cells, showing improved results when compared with biCAR-T cells or single CAR-T cells [256]. More recently, Choi et al. [257] genetically modified CAR-T cells targeting EGFR to deliver bispecific antibodies (bispecific T-cell engager – BiTE) to deal with the GBM heterogeneity. These BiTE-expressing CAR-T cells succeeded in the recruitment of bystander T cells and the elimination of heterogeneous tumors, while avoiding toxicity in normal tissues [74].

Preliminary studies in CAR-T cells showed promising results and bi-, tri- or BiTE CAR-T cells may be an encouraging strategy for the dilemma of antigen loss. Moreover, they have the advantage of exploiting cell killing mechanisms of immune system and of targeting specific antigens of the tumor cells [247]. However, CAR-T cells have limited brain penetration, which implies the use of invasive delivery approaches, and are an individualized product, which renders the treatment expensive [247] when compared to TKI like EGFR inhibitors. Therefore, there are still challenges that have to be overcome. New investigations are needed in order to increase the progresses in immunotherapy in the treatment of GBM patients.

## 5. Conclusions and Future Perspectives

This paper highlights key studies and therapeutics that have marked the investigation and management of gliomas in the past years. Incredible progress has been made and has translated into the development of disruptive and promising therapeutic strategies. Nevertheless, failures are undeniable, pointing to the need for further research in multiple directions. In fact, a better understanding of the complexity of the multifactorial disease and of the interindividual variability is urgently required. Development and implementation of suitable experimental systems that encompass the crosstalk between malignant and host cells and allow personalized drug testing are also needed. Moreover, the development of BBB-permeant drug-delivery systems specifically targeting malignant cells is essential to assure therapeutic concentrations at the target cells, while avoiding toxicity. Hopefully, achievements in the years to come will provide advances beyond the current state-of-the-art in the therapeutics that may be offered to glioma patients, with great impact on survival and quality of life.

## Data Availability

Not applicable.
